# Ornamental birds: hidden carriers of potentially virulent and antimicrobial-resistant *Enterococcus faecalis* in Bangladesh

**DOI:** 10.1128/spectrum.01974-25

**Published:** 2025-11-12

**Authors:** Sadia Afrin Punom, Md. Saiful Islam, Naeem Ahammed Ibrahim Fahim, Md Abdullah Evna Hasan, Md. Tabeer Hossain Antor, Sukumar Saha, Md. Bahanur Rahman, Md. Tanvir Rahman

**Affiliations:** 1 Department of Microbiology and Hygiene, Faculty of Veterinary Science, Bangladesh Agricultural University 54492, Mymensingh, Bangladesh; 2 Department of Animal Science, University of California–Davis, Davis, California, USA; University of Valencia, Paterna, Valencia, Spain

**Keywords:** antibiotic resistance, *Enterococcus faecalis*, ornamental birds, animal feed, multidrug resistance, virulence genes

## Abstract

*Enterococcus faecalis,* a nosocomial pathogen adept at acquiring virulence and resistance genes, may be silently carried by ornamental birds in proximity to humans, posing a health risk. Two hundred ornamental bird samples (feces = 120, cage feed = 60, and store feed = 20) were collected and processed to detect *E. faecalis* isolates and determine their biofilm-forming capabilities, virulence, and antimicrobial resistance profiles. In PCR, 44 (22%) samples were found positive for *E. faecalis*, with the highest occurrence in cage feed samples (18/60), followed by feces (25/120) and stored feed (1/20) samples. Among them, nine *E. faecalis* isolates were determined as strong, 23 were intermediate, and 12 were non-biofilm formers in the Congo Red Agar test. Most isolates carried the following tested virulence genes: *gelE* (95.5%), *fsrC* (93.2%), *ace* (90.9%), *sprE* (88.6%), *fsrB* (88.64%), *fsrA* (81.8%), *pil* (79.5%), *agg* (72.7%), and *cyl* (31.8%). Thirteen *E. faecalis* isolates (29.54%) were phenotypically multidrug-resistant (MDR), including resistance to the reserve group (fosfomycin, 4.5% and linezolid, 11.4%), watch group (vancomycin, 2.3%; ciprofloxacin, 4.5%; and rifampicin, 29.5%), and active group (ampicillin, 100%, erythromycin, 29.5%, and tetracycline, 27.3%). Moreover, all the ampicillin-resistant *E. faecalis* isolates harbored *bla*_TEM_, and 81.8% of tetracycline-resistant isolates contained *tetA* resistance genes. The multiple antibiotic resistance indices of MDR isolates ranged from 0.27 to 0.64. Our findings indicate that ornamental birds and their associated feed can harbor *E. faecalis* with virulence and resistance genes, highlighting the urgent need for strict monitoring, responsible antibiotic use, and improved hygiene to minimize antimicrobial resistance.

**IMPORTANCE:**

Ornamental birds are increasingly popular as pets and companions; however, their role in the spread of antibiotic-resistant and virulent bacteria remains poorly understood. This study reveals that *Enterococcus faecalis*, a bacterium commonly associated with hospital-acquired infections, can be found in ornamental birds and their feed in Bangladesh. Many of the detected strains carried genes that make them more harmful to humans and resistant to multiple antibiotics, including those considered last-resort treatments. These findings are significant because ornamental birds often live in close contact with humans, creating potential pathways for bacterial transmission. The results underscore the need for improved hygiene, responsible antibiotic use in animal husbandry, and regular monitoring of antimicrobial resistance in non-traditional animal species. This research provides crucial insights for public health, particularly in regions where ornamental bird keeping is prevalent but often lacks regulation for microbial safety.

## INTRODUCTION

Enterococci are gram-positive, facultative anaerobic bacteria with a wide geographic distribution (1). They are among the most prevalent organisms in the gastrointestinal tracts of birds and mammals and are routinely recovered from a variety of human infections, including burn wounds, bacteremia, endocarditis, and urinary tract infections, underscoring their clinical importance (2). Within a genus comprising numerous species, *Enterococcus faecalis* and *Enterococcus faecium* remain the most intensively studied. *Enterococcus faecalis* is frequently implicated in endodontic and other odontogenic infections (3), whereas *E. faecium* belongs to the "ESKAPE" pathogens (*E. faecium, Staphylococcus aureus, Klebsiella pneumoniae, Acinetobacter baumannii, Pseudomonas aeruginosa*, and *Enterobacter* spp.), which are responsible for a large proportion of nosocomial infections and are singled out by the World Health Organization (WHO) as priority organisms for new antimicrobials because of their capacity to evade conventional drugs (4).

Ornamental birds rank as the third most common companion animal after dogs and cats, living in close contact with humans (5). Caged birds are dominated by two orders, Psittaciformes (parrots, parakeets, and lovebirds) and Passeriformes (finches and canaries) (6); yet only a handful of studies have examined enterococcal carriage in these species (7–10). For example, Soares et al. (9) reported a high prevalence of *E. faecalis* in pet-bird feed. Because *E. faecalis* can transmit bidirectionally between birds and people, its presence in ornamental birds represents a potential zoonotic risk.

Companion birds have emerged as reservoirs of antibiotic-resistant microorganisms (11). In *Enterococcus*, a rich repertoire of mobile genetic elements, for example, conjugative plasmids, pheromone-responsive plasmids, integrative conjugative elements, and prophages, facilitates inter- and intraspecies gene exchange, blurring the line between commensal and pathogenic species (10). Antimicrobial resistance (AMR) in *E. faecalis* has increased sharply since 2000 (12). Antimicrobial resistance in *Enterococcus* species has become a widespread concern worldwide, with increasing reports of resistance to multiple antibiotic classes, including glycopeptides, penicillins, carbapenems, fluoroquinolones, aminoglycosides, amphenicols, tetracyclines, rifamycins, and others (13). These patterns contribute to the frequent emergence of multidrug-resistant (MDR) strains in diverse ecological niches, including animals, the environment, and human clinical settings. Moreover, biofilm formation magnifies both virulence and resistance, driven by genes and other microbial surface components recognizing adhesive matrix molecules (14). Several virulence genes encode factors involved in adhesion (*ace*, *agg*), biofilm formation (*gelE*, *fsrA/B/C*), immune evasion (*sprE*), or cytotoxicity (*cyl*) (15), suggesting a well-equipped arsenal for host colonization and infection. Strong biofilm-forming isolates exhibited a higher frequency of *agg*, *sprE*, *fsrC*, and *gelE*, all of which have been previously linked to the biofilm matrix structure and quorum sensing. The *fsr* quorum-sensing system is known to regulate the expression of gelatinase (*gelE*) and serine protease (*sprE*), both critical to biofilm maturation and dispersal. Thus, the co-occurrence of these genes in strong biofilm formers may reflect an integrated regulatory mechanism that enhances environmental adaptability and persistence (16). This capacity for resistance and biofilm formation not only complicates treatment but also poses a significant public health concern, given the potential for transmission of resistant and biofilm-forming strains between animals, humans, and the environment.

Enterococci have been reported in fish, migrating birds, shellfish, wild animals, and dairy animals in Bangladesh (17–21), but the virulence and AMR traits of *E. faecalis* from ornamental birds remain uncharacterized. The ornamental-bird sector is expanding in major cities in Bangladesh (22); however, no data exist on the virulence and AMR patterns of *E. faecalis* carried by these birds. This gap is noteworthy because clinically important vancomycin-resistant enterococci (VRE) have been identified in clinical settings (23), and there is limited regulatory oversight of antimicrobial use in the animal sector, including companion birds (24). This study, therefore, aimed to determine the prevalence, virulence-gene profile, and antibiotic susceptibility of *E. faecalis* isolated from ornamental birds to clarify their potential role in enterococcal dissemination.

## MATERIALS AND METHODS

### Sample collection and processing

Two hundred samples were collected from various bird species in retail bird stores across different areas of Mymensingh Sadar Upazila, Bangladesh. These samples included 120 fecal samples, 60 cage feed samples, and 20 store feed samples. In the study, the most common species were budgerigar (*Melopsittacus undulatus*), cockatiel (*Nymphicus hollandicus*), Java sparrow (*Lonchura oryzivora*), finch (*Chlorophonia pyrrhophry*), diamond dove (*Geopelia cuneata*), pigeon (*Columba livia*), parrot (*Psittacula eupatria*), love bird (*Agapornis fischeri*), conure (*Pyrrhura molinae*), and munia (*Lonchura punctulata*). Samples were collected from 20 bird stores, selecting three cages per store to obtain two fecal and one cage feed sample per cage, along with one stored feed sample from each store. Fecal samples were obtained by gently swirling a sterile cotton swab in fresh fecal matter, then transferring the swab into sterile, zip-locked bags labeled with unique identification codes. Cage feed samples (5 g each) were aseptically collected using sterile scoops from feeders within individual cages. Store feed samples were obtained as composite samples from bulk storage containers or feed bags using sterile techniques, and all samples were transferred to sterile, labeled bags for transport. By maintaining a cool chain, all the collected samples were immediately transferred to the Department of Microbiology and Hygiene laboratory at Bangladesh Agricultural University, Mymensingh. Each sample, containing a sterile cotton swab and 1 g of each feed sample, was individually inoculated into 5  mL of sterile nutrient broth (HiMedia, India) and incubated overnight at 37°C in a shaker incubator to facilitate bacterial enrichment.

### Isolation and molecular identification of *E. faecalis*

A loopful of the cultured sample was streaked onto an Enterococcus agar base (EAB) plate (HiMedia, Mumbai, Maharashtra, India) and then incubated for 24 h at 37°C. Colony characteristics on EAB plates (white or pink), staining, and biochemical features (positive for pyrrolidonyl aminopeptidase and negative for catalase) were used to isolate *E. faecalis* (25). Isolates were stored in 50% glycerol for further testing. For molecular detection, the genomic DNA was extracted by the boiling method as described earlier (26). The polymerase chain reaction (PCR) was performed to detect the presence of *E. faecalis* using primers targeting the *ddl_E. faecalis_* gene (Table S1). PCR-positive controls consisted of *E. faecalis* genomic DNA previously confirmed (17) to carry the target genes, whereas negative controls were prepared using non-template reactions, replacing genomic DNA with sterile phosphate-buffered solution (PBS) to detect any potential contamination. One isolate was selected from each sample for PCR analysis.

### The biofilm-forming ability of *E. faecalis*

The Congo Red (CR) agar test was used to qualitatively assess the biofilm development in *E. faecalis* isolates. In the CR test, isolates exhibiting dry, filamentous, crusty black colonies were classified as strong biofilm producers; those forming pink colonies with a dark center were considered intermediate producers; whereas isolates displaying smooth pink colonies were categorized as non-biofilm producers (27, 28).

### Detection of virulence genes

The presence of virulence-associated genes in *E. faecalis*, such as *pil, ace, agg, fsrA, fsrB, fsrC, gelE, sprE*, and *cyl*, was determined using a simplex PCR assay (Table S1). Furthermore, the PCR-positive controls contained *E. faecalis* genomic DNA that had previously tested positive for the particular virulence genes (29). Genomic DNA was substituted with PBS, whereas non-DNA templates served as PCR-negative controls.

### Antibiotic susceptibility testing

Antibiotic susceptibility of *E. faecalis* isolates was assessed using the disk diffusion method (30) in accordance with the 2024 Clinical and Laboratory Standards Institute (CLSI) guidelines (31). A sterile 0.85% normal saline solution was prepared, and following 24 h incubation at 37°C, 2–3 bacterial colonies were suspended in the saline and adjusted to a turbidity equivalent to 0.5 McFarland standard. Sterile cotton swabs were then used to spread the bacterial inoculum onto Mueller-Hinton agar plates. Subsequently, the agar plates were treated with specific antibiotics. Eleven commonly accessible antibiotics from 10 different antibiotic groups were selected, with consideration given to the World Health Organization’s (WHO) Access, Watch, and Reserve (AWaRe) classification. Isolates that demonstrated resistance to at least three antibiotic classes were classified as multidrug-resistant (MDR) (32). The multiple antibiotic resistance (MAR) indices were determined by using the following formula (33):

MAR index = $\frac{the\;count\;of\;antibiotics\;to\;which\;an\;isolates\;showed\;resistance\;}{the\;total\;number\;of\;antibiotics\;employed\;in\;this\;study}$

Genotypic analysis of antimicrobial resistance gene detection was performed to identify the ampicillin-resistant gene, *bla*_TEM_, and the tetracycline-resistant gene, *tetA*, using the method described in Table S1.

### Statistical analysis

The investigation’s data were entered into Excel 365 (Microsoft/Office 365, Redmond, WA, USA) and then exported to SPSS v.25.0 (IBM, Chicago, IL, USA) and GraphPad Prism v.8.4.2 (San Diego, CA, USA) for analysis. Descriptive analysis determined *E. faecalis*-associated prevalence, with a binomial 95% confidence interval (CI_95_) computed using GraphPad Prism. The χ^2^ test was used to identify statistical differences in isolate frequencies and relationships between antibiotic resistance, virulence genes, and biofilm formation. A statistically significant *P*-value was ≤ 0.05. Bivariate analysis in SPSS was used to determine the relationships between antibiotic resistance and virulence genes found in isolates of *E. faecalis*.

## RESULTS

### Prevalence of *E. faecalis*

Out of 200 samples, 44 (22%) samples were found positive in PCR. The highest prevalence was found in cage feed samples (30%) compared with feces (20.8%) and stored feed samples (5%) (*P* > 0.05) (Table 1).

**TABLE 1:** Prevalence of *E. faecalis* isolated from ornamental birds in Mymensingh Sadar, Bangladesh^*a*^

Type of samples (*N*)	*n* (%) [CI_95_]	*P*-value
Feces (120)	25 (20.8) [14.5–28.9]	0.058
Cage feed (60)	18 (30) [19.9–42.5]	
Stored feed (20)	1 (5) [0.8–23.6]	
Total (200)	44 (22) [16.8–28.2]	

^*a*^ *N* = category-wise sample number, *n* = number of sample-wise positive isolates, and CI_95_ = confidence interval at 95%.

### Prevalence of biofilm formation in *E. faecalis* isolates

In the CR agar test, 20.5% (9/44) of isolates were found to be strong biofilm producers. The highest (52.3%) number of isolates was found to be intermediate biofilm producers (23/44), followed by non-biofilm producers (27.2%, 12/44) (Table 2). There was a statistically significant variance in the frequencies of strong, moderate, and non-biofilm-producing *E. faecalis* isolates (Table 2).

**TABLE 2:** Occurrence of biofilm-forming *E. faecalis* isolated from ornamental birds in Mymensingh Sadar, Bangladesh^*a*^

Nature of biofilm	Occurrence of biofilm formers, *n*^s^ (%) (*N* = 44)	CI_95_	*P*-value
Strong	9^b^ (20.4)	11.15–34.5	0.004
Intermediate	23^b^ (52.3)	37.94–66.25	
Weak	12^a^ (27.3)	16.35–41.85	

^*a*^ Values with different s superscripts (a or b) differ significantly (*P* < 0.05). *N* = total number of isolates sampled by category, *n* = number of isolates positive for the category, and CI_95_ = confidence interval at 95%.

### Virulence genes detection

Nine virulence genes were identified across 44 PCR-positive *E. faecalis* isolates, with most isolates carrying multiple genes. The most frequently detected virulence genes were *gelE* (95.5%; 42/44), *fsrC* (93.2%; 41/44), and *ace* (90.9%; 40/44), followed by *sprE* and *fsrB* (88.6% each), *fsrA* (81.8%), *pil* (79.5%), and *agg* (72.7%), whereas *cyl* was the least prevalent (31.8%; 14/44).

In bivariate analysis, positive and strongly significant correlations were found in the presence of virulence genes *agg* and *cyl* (Spearman correlation coefficient, ρ = 0.418), *sprE* and *fsrC* (ρ = 0.471), and *sprE* and *ace* (ρ = 0.385) in *E. faecalis* isolates. Moreover, moderate-to-low positive and significant correlations were determined between the presence of virulence genes *fsrA* and *fsrC* (ρ = 0.340), *fsrB* and *sprE* (ρ = 0.323) in *E. faecalis* isolates (Table S2).

Regarding biofilm production, strong biofilm former isolates had higher levels of *agg, fsrC, gelE*, and *sprE* genes (100%), whereas intermediate biofilm former isolates had higher levels of *ace* (95.7%), *fsrC* and *gelE* (91.3%), and *sprE* and *fsrA* (87%) (Table 3).

**TABLE 3:** Association in the presence of virulence genes and determination of biofilm formation in *E. faecalis* (*n* = 44) isolated from ornamental birds’ samples in Mymensingh Sadar, Bangladesh^*a*^

Virulence gene names	Virulence in different degrees of biofilm formation			Total no. (%) of positive isolates [CI_95_]	*P*-value
	No. (%) of strong biofilm former (*N* = 9)	No. (%) of intermediate biofilm former (*N* = 23)	No. (%) of non-biofilm former (*N* = 12)		
*ace*	7 (77.8)	22 (95.7)	11 (91.7)	40 (90.9) [78.84–97.47]	0.285
*agg*	9 (100)	14 (60.9)	9 (75)	32 (72.7) [58.15–83.65]	0.081
*cyl*	3 (33.3)	5 (21.7)	6 (50)	14 (31.8) [20–46.56]	0.233
*fsrA*	7 (77.8)	20 (87.0)	9 (75)	36 (81.8) [68.04–90.49]	0.643
*fsrB*	8 (88.9)	19 (82.6)	12 (100)	39 (88.6) [76.02–95.05]	0.306
*fsrC*	9 (100)	21 (91.3)	11 (91.7)	41 (93.2) [81.77–97.65]	0.661
*gelE*	9 (100)	21 (91.3)	12 (100)	42 (95.5) [84.87–98.74]	0.384
*pil*	6 (66.7)	17 (73.9)	12 (100)	35 (79.5) [65.5–88.85]	0.108
*sprE*	9 (100)	20 (87)	10 (83.3)	39 (88.6) [76.02–95.05]	0.460

^*a*^ *N* = total number of isolates tested, and CI_95_ = confidence interval at 95%.

### Antibiotic resistance profiles of *E. faecalis*

Antibiogram analysis of *E. faecalis* isolates (*N* = 44) revealed that within the WHO Access antibiotic group, all isolates were phenotypically resistant to ampicillin, 29.5% were resistant to erythromycin, and 27.3% were resistant to tetracycline; within the Watch group, 29.5% were resistant to rifampicin, 4.5% were resistant to ciprofloxacin, and 2.3% were resistant to vancomycin; and within the Reserve group of antibiotics, 11.4% were resistant to linezolid and 4.5% were resistant to fosfomycin. Moreover, all the ampicillin-resistant isolates contained the resistance gene *bla*_TEM_ (44/44), and 81.81% of the tetracycline-resistant isolates had the *tetA* gene (9/11).

Statistical bivariate analysis revealed that the antibiotic-resistant isolates examined in this study exhibited significant correlations with one another. There was a positive link between the resistance patterns of vancomycin and linezolid (ρ = 0.426), erythromycin and linezolid (ρ = 0.396), vancomycin and nitrofurantoin (ρ = 0.564), erythromycin and nitrofurantoin (ρ = 0.418), vancomycin and fosfomycin (ρ = 0.699), chloramphenicol and ciprofloxacin (ρ = 0.699), tetracycline and ciprofloxacin (ρ = 0.356), chloramphenicol and erythromycin (ρ = 0.337), nitrofurantoin and fosfomycin (ρ = 0.374), fosfomycin and rifampicin (ρ = 0.337), and chloramphenicol and tetracycline (ρ = 0.356) (Table S3).

Moreover, strong biofilm-forming *E. faecalis* isolates had higher resistance patterns than intermediate- and non-biofilm formers, including vancomycin (11.1% vs. 0% vs. 0%), ciprofloxacin (11.1% vs. 4.3% vs. 0%), erythromycin (33.3% vs. 30.4% vs. 25%), fosfomycin (11.1% vs. 4.3% vs. 0%), and nitrofurantoin (11.1% vs. 8.7% vs. 0%) (Table 4).

**TABLE 4:** Relationship between biofilm production and antibiotic resistance patterns in *E. faecalis* isolated from ornamental birds in Mymensingh Sadar, Bangladesh^*a*^

Antibiotics	Biofilm formation level			Total no. (%) of resistant isolates [CI_95_] (*N* = 44)	*P*-value
	No.^s^ of strong biofilm former (*N* = 9)	No.^s^ (%) of intermediate biofilm former (*N* = 23)	No.^s^ (%) of non-biofilm former (*N* = 12)		
AMP	9^a^ (100)	23^a^ (100)	12^a^ (100)	44 (100) [91.97–100]	NA
VAN	1^a^ (11.1)	0^a^ (0)	0^a^ (0)	1 (2.3) [0.40–11.81]	0.137
CIP	1^a^ (11.1)	1^a^ (4.3)	0^a^ (0)	2 (4.5) [1.25–15.13]	0.480
C	0^a^ (0)	2^a^ (8.7)	0^a^ (0)	2 (4.5) [1.25–15.13]	0.384
TE	3^a^ (33.3)	9^a^ (39.1)	0^b^ (0)	12 (27.3) [16.35–41.85]	0.043
TEC	0^a^ (0)	0^a^ (0)	0^a^ (0)	0 (0) [0–8.03]	NA
E	3^a^ (33.3)	7^a^ (30.4)	3^a^ (25)	13 (29.5) [18.16–44.22]	0.909
RA	1^a^ (11.1)	8^a^ (34.8)	4^a^ (33.3)	13 (29.5) [18.16–44.22]	0.396
LNZ	1^a^ (11.1)	2^a^ (8.7)	2^a^ (16.7)	5 (11.4) [4.95–23.98]	0.780
FOS	1^a^ (11.1)	1^a^ (4.3)	0^a^ (00)	2 (4.5) [1.25–15.13]	0.480
NIT	1^a^ (11.1)	2^a^ (8.7)	0^a^ (00)	3 (6.8) [2.34–18.23]	0.531

^*a*^ Values with different s superscripts (a or b) differ significantly (*P* < 0.05). *N* = total number of isolates sampled, CI_95_ = confidence interval at 95%, AMP = ampicillin, CIP = ciprofloxacin, C = chloramphenicol, E = erythromycin, LEV = levofloxacin, LZD = linezolid, NIT = nitrofurantoin, RA = rifampin, TE = tetracycline, TEC = teicoplanin, and VAN = vancomycin.

Thirteen (29.5%) *E. faecalis* isolates exhibited phenotypic multidrug resistance, displaying 10 distinct resistance patterns among them. Moreover, the MAR index of those MDR isolates ranged between 0.27 and 0.64 (Table 5).

**TABLE 5:** The overall occurrence of multidrug-resistant and multiple antibiotic-resistant *E. faecalis* isolated from ornamental birds in Mymensingh Sadar, Bangladesh^*a*^

Serial no.	Antibiotic resistance pattern	No. of antibiotics (classes)	No. of isolates	Overall MDR isolates	MAR index
1	AMP, E, TE, CIP, C	5(5)	1	13/44 (29.5%)	0.45
2	LNZ, AMP, VAN, E, NIT, RA, FOS	7(7)	1		0.64
3	LNZ, AMP, E	3(3)	1		0.27
4	AMP, TE, RA, FOS	4(4)	1		0.36
5	AMP, TE, RA	3(3)	1		0.27
6	AMP, E, TE	3(3)	2		0.27
7	LNZ, AMP, E, RA	4(4)	2		0.36
8	AMP, E, TE, NIT	4(4)	2		0.36
9	AMP, E, TE, C	4(4)	1		0.36
10	AMP, TE, CIP	3(3)	1		0.27

^*a*^ MDR = multidrug-resistant, MAR = multiple antibiotic resistance, AMP = ampicillin, CIP = ciprofloxacin, C = chloramphenicol, E = erythromycin, LEV = levofloxacin, LZD = linezolid, NIT = nitrofurantoin, RA = rifampin, TE = tetracycline, and VAN = vancomycin.

## DISCUSSION

Enterococci, once considered harmless gut commensals of humans and animals, are now major causes of nosocomial infections, with rising antibiotic resistance posing a public health threat (9). However, limited data exist on their resistance patterns in ornamental birds and their feed in Bangladesh. This study aimed to investigate the prevalence of biofilm-forming *Enterococcus* isolates from ornamental birds and their association with virulence genes and antibiotic resistance in Bangladesh.

### Prevalence of *E. faecalis*

In this study, the overall prevalence of *E. faecalis* was 22%, with a higher detection rate in cage feed samples (30%) compared with feces (20.8%). The higher prevalence of *E. faecalis* in cage feed compared with fecal samples may be attributed to direct fecal contamination, as birds often defecate near or into feeders. The nutrient-rich feed can support bacterial survival longer than feces, which may dry out or contain competing microbes (34). Inadequate cleaning of feeders and cross-contamination during handling may further contribute to the persistence and spread of *E. faecalis* in the feeding environment.

Comparable studies have reported varying prevalence rates depending on bird type and sample source. For instance, a study in Poland reported a 39.1% prevalence among free-living birds (35), whereas Soodmand et al. (10) recorded a 25.3% prevalence from cloacal swabs of companion birds in northeastern Iran. In Portugal, Da Silva et al. (36) and Santos et al. (7) isolated 100% and 42% *E. faecalis*, respectively, from cloacal swab samples of pet and exotic birds. Soares et al. (9) also reported a 36.7% prevalence in feed samples from ornamental birds, which is substantially higher than the 5% found in stored feed samples in the present study. Variations in the prevalence of *E. faecalis* across studies may be influenced by differences in bird management, feed source and storage, visitor handling, geographic location, environmental conditions, sample type and size, and regional bacterial load.

The detection of *E. faecalis* in ornamental birds and their associated environments highlights a potential, yet underrecognized, public health concern. Enterococci are natural inhabitants of the gastrointestinal tract in both humans and animals (37); however, their detection in non-clinical settings, such as ornamental bird cages and feed, suggests a broader ecological persistence. Although their presence in avian feces is expected (38), the notable prevalence in cage feed samples suggests possible environmental contamination, likely due to direct fecal contact or poor hygienic practices during feed storage and handling. This raises concerns not only about cross-contamination within bird enclosures but also about the potential for zoonotic transfer, especially in domestic or hobbyist settings where birds are kept in close contact with humans. The relatively lower detection in stored feed compared with cage feed may reflect better hygienic controls at the supplier or manufacturer level, where feeds were typically packaged and stored in sealed bags. In contrast, cage feed was observed to be stored in open containers or left directly in feeders accessible to birds, creating more opportunities for contamination from fecal matter, handling, or the surrounding environment. This difference suggests that contamination is more likely to occur at the point of use, highlighting the need for targeted interventions in feed handling and hygiene practices within pet shops and homes.

### Biofilm-forming capabilities of isolated *E. faecalis*

Biofilm formation is a critical survival strategy that enables *E. faecalis* to persist in hostile environments, evade host immune responses, and resist antimicrobial treatments (39). In this study, a higher percentage of *E. faecalis* isolates (72.7%; 32/44) were biofilm formers, with 20.5% (9/44) classified as strong biofilm formers, 52.3% (23/44) as intermediate, and 27.2% (12/44) as non-biofilm producers. A previous study (40) found that *E. faecalis* isolated from backyard chickens had biofilm-forming capabilities, with 28.9% as strong biofilm-forming *E. faecalis*. Similarly, Stępień-Pyśniak et al. (8) found that 25.9% of *E. faecalis* isolates from wild birds were strong biofilm producers. Although backyard chickens, wild birds, and ornamental birds differ in their husbandry and exposure settings, these findings collectively suggest that biofilm-forming *E. faecalis* is widespread across diverse avian populations beyond commercial poultry systems.

A considerable proportion of isolates exhibited intermediate-to-strong biofilm-forming ability, reinforcing their pathogenic potential even in non-clinical avian hosts. The dominance of intermediate biofilm formers, followed by strong biofilm producers, is particularly concerning given the implications for environmental persistence and chronic colonization. The presence of biofilm-forming *E. faecalis* in ornamental birds suggests that these environments could act as persistent reservoirs, with potential for recurring transmission cycles. This becomes more alarming in pet shops and households with limited biosecurity measures, where persistent colonization may lead to unnoticed spread among birds or to caretakers. Importantly, biofilm-forming capacity is often associated with enhanced virulence and antimicrobial resistance. Previous studies have demonstrated that biofilm-associated cells can exhibit up to 100-fold to 1,000-fold increased tolerance to antibiotics compared with their planktonic counterparts (41). Therefore, even if phenotypic resistance appears moderate, the presence of biofilm-forming strains may lead to treatment failure or prolonged infections (42). Additionally, the heterogeneity in biofilm-forming ability among isolates suggests potential strain-level differences in regulatory pathways, nutrient adaptation, and virulence gene expression, areas that merit further molecular investigation (43).

### Virulence genes in isolated *E. faecalis*

A key finding of this study was the widespread presence of multiple virulence genes among *E. faecalis* isolates, with all the isolates having at least three virulence genes and most strains harboring more than four. The most prevalent were *gelE*, *fsrC*, and *ace*, each detected in over 90% of isolates. Several studies have been done on wild birds, but no results are available specifically for ornamental birds. Sigirci et al. (44) found *gelE* in 30.1% of *E. faecalis* isolates*,* whereas Silva et al. (45) revealed its absence. The presence of *E. faecalis* in ornamental birds observed in our study is consistent with reports from Saudi Arabia and Poland, where the bacterium has also been isolated from backyard chickens, wild birds, and free-living birds (8, 35, 40). This suggests that *E. faecalis* is widely distributed across diverse avian hosts, and although husbandry practices differ, the ability of both ornamental and non-commercial bird populations to harbor this species underscores their potential role as reservoirs of antimicrobial-resistant bacteria.

### Antimicrobial resistance profiles of *E. faecalis*

The antimicrobial susceptibility profile of *E. faecalis* isolates revealed a worrisome pattern of resistance, particularly to commonly used first-line antibiotics. All isolates were resistant to ampicillin, indicating a complete loss of susceptibility to this Access-class drug. Resistance was also observed for tetracycline (27.3%) and erythromycin (29.5%), both frequently used in veterinary and human medicine (46). The detection of *bla*_TEM_ in all ampicillin-resistant isolates and *tetA* in the majority of tetracycline-resistant strains confirms the genetic basis of resistance and suggests the involvement of mobile resistance determinants facilitating horizontal gene transfer. The presence of such genes is particularly concerning, as both *bla*_TEM_ and *tetA* are commonly carried on plasmids or other mobile genetic elements, which enhances their potential for dissemination among bacterial populations (47). This means that resistant *E. faecalis* strains in ornamental birds not only pose a direct risk of treatment failure but may also serve as a reservoir for resistance determinants that can be transferred to other pathogenic or commensal bacteria, amplifying the public health threat. Notably, resistance extended beyond the Access group into the WHO Watch and Reserve categories. Although the prevalence was lower, resistance to rifampicin (29.5%), linezolid (11.4%), vancomycin (2.3%), and fosfomycin (4.5%) is particularly concerning. These antibiotics are critical for treating multidrug-resistant gram-positive infections in humans, and their reduced efficacy in environmental or animal-associated strains highlights the silent spread of clinically significant resistance (48–50). The detection of vancomycin resistance, albeit rare, is especially alarming due to the role of VRE in hospital-acquired infections. Its presence in ornamental birds’ isolates suggests that even non-therapeutically treated populations may acquire resistance genes via environmental exposure or horizontal gene transfer. Previous studies have reported that enterococci isolated from bird samples exhibit varying levels of AMR against a range of antimicrobial agents, as documented in studies from Turkey (44), Malaysia (51), and Iran (10).

Correlative analysis further revealed significant associations between resistance to different antibiotics, particularly among linezolid, vancomycin, fosfomycin, and nitrofurantoin. These co-resistance patterns may reflect co-selection or co-localization of resistance genes on plasmids or transposons. Such cross-resistance complicates empirical therapy and may lead to treatment failure if unnoticed. Importantly, strong biofilm-forming isolates exhibited higher resistance frequencies for several antibiotics, including vancomycin, ciprofloxacin, erythromycin, and nitrofurantoin. Although not all associations reached statistical significance, biofilm-producing *E. faecalis* often exhibits enhanced resistance due to limited drug penetration and altered metabolic states within the biofilm matrix (52). The biofilm matrix restricts antibiotic penetration and facilitates gene exchange, thereby creating a niche for the survival of MDR strains (52). This finding emphasizes the importance of assessing biofilm capacity when evaluating antimicrobial susceptibility, as standard planktonic MIC testing may underestimate resistance *in vivo*.

Additionally, over one-fourth of the isolates (29.5%) were classified as MDR. These MDR isolates exhibited diverse resistance patterns, with MAR index values ranging from 0.27 to 0.64, indicating higher antimicrobial exposure or selection pressure. Although ornamental birds are not routinely exposed to antimicrobials, the presence of MDR strains in their environment suggests indirect exposure through contaminated feed, water, or human handlers. Importantly, some MDR patterns included resistance to as many as seven different classes, illustrating the genomic plasticity and resilience of *E. faecalis*. The emergence of such complex resistance profiles in an ornamental bird setting, a niche not traditionally associated with antimicrobial misuse, highlights the diffuse nature of resistance evolution and the need for One Health-based surveillance strategies.

### Conclusion

This first study, to the best of our knowledge, in Bangladesh provides an important insight into the potential public health risks associated with *E. faecalis* in ornamental birds and their environment. The investigation highlights that ornamental birds can harbor *E. faecalis* isolates with antimicrobial resistance, biofilm-forming capacity, and virulence-associated genes, suggesting their potential role as reservoirs of traits that may contribute to pathogenicity and resistance dissemination, with possible implications for both animal and human health. As ornamental birds are increasingly kept as pets and traded in close proximity to humans, their role in the transmission of opportunistic pathogens warrants attention. These findings underscore the importance of enhanced biosecurity, routine microbial surveillance, and prudent antimicrobial stewardship in non-clinical settings. A One Health approach that integrates animal, environmental, and human health perspectives is essential to mitigate the emergence and spread of antimicrobial resistance. This study lays the groundwork for future research and policy development aimed at safeguarding public health through a deeper understanding and more effective management of zoonotic risks in the ornamental bird industry in Bangladesh.

## Data Availability

All the data are presented in the article and the supplemental files.

## SUPPLEMENTAL MATERIAL

Supplemental material spectrum.01974-25-s0001.xlsx Raw data.

Supplemental tables spectrum.01974-25-s0002.docx Tables S1 to S3.

ASM does not own the copyrights to Supplemental Material that may be linked to, or accessed through, an article. The authors have granted ASM a non-exclusive, world-wide license to publish the Supplemental Material files. Please contact the corresponding author directly for reuse.

## References

[1] Kense MJ, Landman WJM. 2011. Enterococcus cecorum infections in broiler breeders and their offspring: molecular epidemiology. Avian Pathol 40:603–612. doi:10.1080/03079457.2011.619165 22107095

[2] Vankerckhoven V, Huys G, Vancanneyt M, Vael C, Klare I, Romond M-B, Entenza JM, Moreillon P, Wind RD, Knol J, Wiertz E, Pot B, Vaughan EE, Kahlmeter G, Goossens H. 2008. Biosafety assessment of probiotics used for human consumption: recommendations from the EU-PROSAFE project. Trends Food Sci Technol 19:102–114. doi:10.1016/j.tifs.2007.07.013

[3] Anderson AC, Jonas D, Huber I, Karygianni L, Wölber J, Hellwig E, Arweiler N, Vach K, Wittmer A, Al-Ahmad A. 2015. Enterococcus faecalis from food, clinical specimens, and oral sites: prevalence of virulence factors in association with biofilm formation. Front Microbiol 6:1534. doi:10.3389/fmicb.2015.01534 26793174 PMC4707231

[4] Rice LB. 2008. Federal funding for the study of antimicrobial resistance in nosocomial pathogens: no ESKAPE, p 1079–1081. The University of Chicago Press. 10.1086/533452 18419525

[5] Cong W, van Ruijven J, Mommer L, De Deyn GB, Berendse F, Hoffland E. 2014. Plant species richness promotes soil carbon and nitrogen stocks in grasslands without legumes. Journal of Ecology 102:1163–1170. doi:10.1111/1365-2745.12280

[6] Boseret G, Losson B, Mainil JG, Thiry E, Saegerman C. 2013. Zoonoses in pet birds: review and perspectives. Vet Res 44:1–17. doi:10.1186/1297-9716-44-36 23687940 PMC3668993

[7] Santos T, Silva N, Igrejas G, Rodrigues P, Micael J, Rodrigues T, Resendes R, Gonçalves A, Marinho C, Gonçalves D, Cunha R, Poeta P. 2013. Dissemination of antibiotic resistant Enterococcus spp. and Escherichia coli from wild birds of azores archipelago. Anaerobe 24:25–31. doi:10.1016/j.anaerobe.2013.09.004 24047647

[8] Stępień-Pyśniak D, Hauschild T, Kosikowska U, Dec M, Urban-Chmiel R. 2019. Biofilm formation capacity and presence of virulence factors among commensal Enterococcus spp. from wild birds. Sci Rep 9:11204. doi:10.1038/s41598-019-47602-w 31371744 PMC6671946

[9] Soares R, Miranda C, Cunha S, Ferreira L, Martins Â, Igrejas G, Poeta P. 2023. Antibiotic resistance of Enterococcus species in ornamental animal feed. Animals (Basel) 13:1761. doi:10.3390/ani13111761 37889631 PMC10251925

[10] Soodmand J, Zeinali T, Kalidari G, Hashemitabar G, Razmyar J. 2018. Antimicrobial susceptibility profile of Enterococcus species isolated from companion birds and poultry in the Northeast of Iran. Arch Razi Inst 73:207–213. doi:10.22092/ari.2017.108332.1089 30280840

[11] Marinho CM, Santos T, Gonçalves A, Poeta P, Igrejas G. 2016. A decade-long commitment to antimicrobial resistance surveillance in Portugal. Front Microbiol 7:1650. doi:10.3389/fmicb.2016.01650 27843438 PMC5086874

[12] Guan L, Beig M, Wang L, Navidifar T, Moradi S, Motallebi Tabaei F, Teymouri Z, Abedi Moghadam M, Sedighi M. 2024. Global status of antimicrobial resistance in clinical Enterococcus faecalis isolates: systematic review and meta-analysis. Ann Clin Microbiol Antimicrob 23:80. doi:10.1186/s12941-024-00728-w 39182092 PMC11344933

[13] Huang C, Moradi S, Sholeh M, Tabaei FM, Lai T, Tan B, Meng J, Azizian K. 2025. Global trends in antimicrobial resistance of Enterococcus faecium: a systematic review and meta-analysis of clinical isolates. Front Pharmacol 16:1505674. doi:10.3389/fphar.2025.1505674 40260375 PMC12009923

[14] Fahim N, Masud R, Salam S, Hasan M, Rahman A, Punom S. 2025. Role of Enterococcus in spreading antimicrobial resistance genes and its public health significance. Ger J Vet Res 5:95–112. doi:10.51585/gjvr.2025.1.0123

[15] Hashem YA, Amin HM, Essam TM, Yassin AS, Aziz RK. 2017. Biofilm formation in enterococci: genotype-phenotype correlations and inhibition by vancomycin. Sci Rep 7:5733. doi:10.1038/s41598-017-05901-0 28720810 PMC5515943

[16] Jia J, Liu Q, Zhao E, Li X, Xiong X, Wu C. 2024. Biofilm formation on microplastics and interactions with antibiotics, antibiotic resistance genes and pathogens in aquatic environment. Eco-Environ Health 3:516–528. doi:10.1016/j.eehl.2024.05.003 39605964 PMC11599983

[17] Ferdous FB, Islam MS, Ullah MA, Rana ML, Punom SA, Neloy FH, Chowdhury MNU, Hassan J, Siddique MP, Saha S, Rahman MT. 2023. Antimicrobial resistance profiles, virulence determinants, and biofilm formation in enterococci isolated from rhesus macaques (Macaca mulatta): a potential threat for wildlife in Bangladesh? Animals (Basel) 13:2268. doi:10.3390/ani13142268 37508046 PMC10376288

[18] Saiful Islam Md, Paul A, Talukder M, Roy K, Abdus Sobur Md, Ievy S, Mehedi Hasan Nayeem Md, Rahman S, Nazmul Hussain Nazir KHM, Tofazzal Hossain M, Tanvir Rahman Md. 2021. Migratory birds travelling to Bangladesh are potential carriers of multi-drug resistant Enterococcus spp., Salmonella spp., and Vibrio spp. Saudi J Biol Sci 28:5963–5970. doi:10.1016/j.sjbs.2021.06.053 34588913 PMC8459117

[19] Fahim NAI, Rana ML, Hasan MAE, Salam S, Masud RI, Huda N, Saha S, Rahman MT. 2025. Biofilms and antibiotic resistance profile of Enterococcus faecalis in selected dairy cattle farm environments in Bangladesh. PLoS One 20:e0323667. doi:10.1371/journal.pone.0323667 40388390 PMC12087997

[20] Ullah MA, Islam MS, Ferdous FB, Rana ML, Hassan J, Rahman MT. 2024. Assessment of prevalence, antibiotic resistance, and virulence profiles of biofilm-forming Enterococcus faecalis isolated from raw seafood in Bangladesh. Heliyon 10:e39294. doi:10.1016/j.heliyon.2024.e39294 39640770 PMC11620263

[21] Rana ML, Firdous Z, Ferdous FB, Ullah MA, Siddique MP, Rahman MT. 2023. Antimicrobial resistance, biofilm formation, and virulence determinants in Enterococcus faecalis isolated from cultured and wild fish. Antibiotics (Basel) 12:1375. doi:10.3390/antibiotics12091375 37760672 PMC10525749

[22] Yasmin N, Khan MA, Mortuza MG. 2024. An account of ornamental bird status and species assemblage in the bird shop of Rajshahi City Corporation, Bangladesh. IJEAB 9:083–087. doi:10.22161/ijeab.93.9

[23] Roy S, Aung MS, Paul SK, Khan MdNA, Nasreen SA, Hasan MS, Haque N, Barman TK, Khanam J, Sathi FA, Paul S, Ali MI, Kobayashi N. 2025. Isolation of vanA-mediated vancomycin-resistant Enterococcus faecalis (ST1912/CC116) and Enterococcus faecium (ST80/CC17), optrA-positive linezolid-resistant E. faecalis (ST32, ST1902) from human clinical specimens in Bangladesh. Antibiotics (Basel) 14:261. doi:10.3390/antibiotics14030261 40149072 PMC11939402

[24] Al Amin M, Hoque MN, Siddiki AZ, Saha S, Kamal MM. 2020. Antimicrobial resistance situation in animal health of Bangladesh. Vet World 13:2713–2727. doi:10.14202/vetworld.2020.2713-2727 33487990 PMC7811541

[25] Manero A, Blanch AR. 1999. Identification of Enterococcus spp. with a biochemical key. Appl Environ Microbiol 65:4425–4430. 10508070 10.1128/aem.65.10.4425-4430.1999 PMC91588

[26] Mahmud S, Nazir KHMNH, Rahman MdT. 2018. Prevalence and molecular detection of fluoroquinolone-resistant genes (qnrA and qnrS) in Escherichia coli isolated from healthy broiler chickens. Vet World 11:1720–1724. doi:10.14202/vetworld.2018.1720-1724 30774264 PMC6362325

[27] Souza EL de, Meira QGS, Barbosa I de M, Athayde AJAA, Conceição ML da, Siqueira Júnior JP de. 2014. Biofilm formation by Staphylococcus aureus from food contact surfaces in a meat-based broth and sensitivity to sanitizers. Braz J Microbiol 45:67–75. doi:10.1590/S1517-83822014000100010 24948915 PMC4059327

[28] Ballah FM, Islam MS, Rana ML, Ferdous FB, Ahmed R, Pramanik PK, Karmoker J, Ievy S, Sobur MA, Siddique MP, Khatun MM, Rahman M, Rahman MT. 2022. Phenotypic and genotypic detection of biofilm-forming Staphylococcus aureus from different food sources in Bangladesh. Biology (Basel) 11:949. doi:10.3390/biology11070949 36101330 PMC9311614

[29] Ullah MA, Islam MS, Rana ML, Ferdous FB, Neloy FH, Firdous Z, Hassan J, Rahman MT. 2023. Resistance profiles and virulence determinants in biofilm-forming Enterococcus faecium isolated from raw seafood in Bangladesh. Pathogens 12:1101. doi:10.3390/pathogens12091101 37764909 PMC10535238

[30] Bauer A. 1996. Antibiotic susceptibility testing by a standardized single disc method. Am J of Clinc Path 45:149–158.

[31] Clinical and Laboratory Standards Institute. 2024. Performance standards for antimicrobial testing. In CLSI supplement M100, 34th ed. Clinical and Laboratory Standards Institute, Wayne, PA.

[32] Magiorakos A-P, Srinivasan A, Carey RB, Carmeli Y, Falagas ME, Giske CG, Harbarth S, Hindler JF, Kahlmeter G, Olsson-Liljequist B, Paterson DL, Rice LB, Stelling J, Struelens MJ, Vatopoulos A, Weber JT, Monnet DL. 2012. Multidrug-resistant, extensively drug-resistant and pandrug-resistant bacteria: an international expert proposal for interim standard definitions for acquired resistance. Clin Microbiol Infect 18:268–281. doi:10.1111/j.1469-0691.2011.03570.x 21793988

[33] Krumperman PH. 1983. Multiple antibiotic resistance indexing of Escherichia coli to identify high-risk sources of fecal contamination of foods. Appl Environ Microbiol 46:165–170. doi:10.1128/aem.46.1.165-170.1983 6351743 PMC239283

[34] Meyers BC, McLellan SL. 2022. Influence of nutrients and the native community on E. coli survival in the beach environment. Appl Environ Microbiol 88:e01043-22. doi:10.1128/aem.01043-22 36218359 PMC9642020

[35] Kwit R, Zając M, Śmiałowska-Węglińska A, Skarżyńska M, Bomba A, Lalak A, Skrzypiec E, Wojdat D, Koza W, Mikos-Wojewoda E, Pasim P, Skóra M, Polak M, Wiącek J, Wasyl D. 2023. Prevalence of Enterococcus spp. and the whole-genome characteristics of Enterococcus faecium and Enterococcus faecalis strains isolated from free-living birds in Poland. Pathogens 12:836. doi:10.3390/pathogens12060836 37375526 PMC10305306

[36] da Silva B da CT, de Carvalho DUOG, Sakauchi VTS, Ferreira JS, Cortez A, Heinemann MB, Gaeta NC. 2024. Investigating antimicrobial-resistant bacteria from exotic domestic birds - a one health concern. Braz J Vet Med 46:e001624. doi:10.29374/2527-2179.bjvm001624 39119241 PMC11308690

[37] Cattoir V. 2022. The multifaceted lifestyle of enterococci: genetic diversity, ecology and risks for public health. Curr Opin Microbiol 65:73–80. doi:10.1016/j.mib.2021.10.013 34768106

[38] Golden CE, Rothrock MJ Jr, Mishra A. 2021. Mapping foodborne pathogen contamination throughout the conventional and alternative poultry supply chains. Poult Sci 100:101157. doi:10.1016/j.psj.2021.101157 34089937 PMC8182426

[39] Mohamed JA, Huang DB. 2007. Biofilm formation by enterococci. J Med Microbiol 56:1581–1588. doi:10.1099/jmm.0.47331-0 18033823

[40] Alzahrani OM, Fayez M, Alswat AS, Alkafafy M, Mahmoud SF, Al-Marri T, Almuslem A, Ashfaq H, Yusuf S. 2022. Antimicrobial resistance, biofilm formation, and virulence genes in Enterococcus species from small backyard chicken flocks. Antibiotics (Basel) 11:380. doi:10.3390/antibiotics11030380 35326843 PMC8944505

[41] Høiby N, Bjarnsholt T, Givskov M, Molin S, Ciofu O. 2010. Antibiotic resistance of bacterial biofilms. Int J Antimicrob Agents 35:322–332. doi:10.1016/j.ijantimicag.2009.12.011 20149602

[42] Gajewska J, Chajęcka-Wierzchowska W, Byczkowska-Rostkowska Z, Saki M. 2023. Biofilm formation capacity and presence of virulence determinants among Enterococcus species from milk and raw milk cheeses. Life (Basel) 13:495. doi:10.3390/life13020495 36836852 PMC9962698

[43] Mould DL, Hogan DA. 2021. Intraspecies heterogeneity in microbial interactions. Curr Opin Microbiol 62:14–20. doi:10.1016/j.mib.2021.04.003 34034081 PMC8459320

[44] Diren Sigirci B, Celik B, Halac B, Basaran Kahraman B, Bagcigil A, Ak S. 2021. Characterization of faecal enterococci from wild birds in turkey and its importance in antimicrobial resistance. J Hellenic Vet Med Soc 72:3015. doi:10.12681/jhvms.28482

[45] Silva N, Igrejas G, Rodrigues P, Rodrigues T, Gonçalves A, Felgar AC, Pacheco R, Gonçalves D, Cunha R, Poeta P. 2011. Molecular characterization of vancomycin-resistant enterococci and extended-spectrum β-lactamase-containing Escherichia coli isolates in wild birds from the azores archipelago. Avian Pathol 40:473–479. doi:10.1080/03079457.2011.599061 21834624

[46] European Food Safety Authority (EFSA), European Centre for Disease Prevention and Control (ECDC). 2023. The European Union Summary Report on Antimicrobial Resistance in zoonotic and indicator bacteria from humans, animals and food in 2020/2021. EFS2 21:e07867. doi:10.2903/j.efsa.2023.7867 PMC9987209 36891283

[47] Kumavath R, Gupta P, Tatta ER, Mohan MS, Salim SA, Busi S. 2025. Unraveling the role of mobile genetic elements in antibiotic resistance transmission and defense strategies in bacteria. Front Syst Biol 5:1557413. doi:10.3389/fsysb.2025.1557413 40810119 PMC12342005

[48] Schwalm JD, El-Helou P, Lee CH. 2004. Clinical outcome with oral linezolid and rifampin following recurrent methicillin-resistant Staphylococcus aureus bacteremia despite prolonged vancomycin treatment. Can J Infect Dis 15:97–100. doi:10.1155/2004/768765 18159483 PMC2094963

[49] Hourigan D, Stefanovic E, Hill C, Ross RP. 2024. Promiscuous, persistent and problematic: insights into current enterococcal genomics to guide therapeutic strategy. BMC Microbiol 24:103. doi:10.1186/s12866-024-03243-2 38539119 PMC10976773

[50] Oliva A, Stefani S, Venditti M, Di Domenico EG. 2021. Biofilm-related infections in gram-positive bacteria and the potential role of the long-acting agent dalbavancin. Front Microbiol 12:749685. doi:10.3389/fmicb.2021.749685 34745053 PMC8569946

[51] Leong SS, Lihan S, Chia HC. 2020. Antibiotic resistance and virulence genes detection among the Enterococcus faecalis isolated from swiftlet industry in borneo. ijph 28:673–687. doi:10.18502/ijph.v49i6.3372

[52] Uruén C, Chopo-Escuin G, Tommassen J, Mainar-Jaime RC, Arenas J. 2020. Biofilms as promoters of bacterial antibiotic resistance and tolerance. Antibiotics (Basel) 10:3. doi:10.3390/antibiotics10010003 33374551 PMC7822488

