# Supplementary material for: Ornamental birds: hidden carriers of potentially virulent and antimicrobial-resistant Enterococcus faecalis in Bangladesh
Source: Microbiol Spectr. 2025 Nov 12;13(12):e01974-25. doi: 10.1128/spectrum.01974-25 (PMC12671201; doi:10.1128/spectrum.01974-25)
Supplement: Supplemental tables — Tables S1 to S3. [file spectrum.01974-25-s0002.docx]

| **Targeted factors** | **Targeted genes** | **Primer Sequences (5′-3′)** | **Annealing Tm (° C)** | **Size (bp)** | **References** |
| --- | --- | --- | --- | --- | --- |
| *E. faecalis* | *ddl_E. faecalis_* | F: ATCAAGTACAGTTAGTCTT | 50 | 941 | (1) |
|  |  | R: ACGATTCAAAGCTAACTG |  |  |  |
| Virulence genes | *ace* | F: GAATGACCGAGAACGATGGC | 58 | 615 | (2) |
|  |  | R: CTTGATGTTGGCCTGCTTCC |  |  |  |
|  | *agg* | F: TCTTGGACACGACCCATGAT | 58 | 413 | (2) |
|  |  | R:AGAAAGAACATCACCACGAGC |  |  |  |
|  | *cyl* | F: TGGCGGTATTTTTACTGGAG | 52 | 186 | (3) |
|  |  | R: TGAATCGCTCCATTTCTTC |  |  |  |
|  | *fsrA* | F: CGTTCCGTCTCTCATAGTTA | 53 | 474 | (2) |
|  |  | R: GC*AGG*ATTTG*AGG*TTGCTAA |  |  |  |
|  | *fsrB* | F: TAATCT*AGG*CTTAGTTCCCAC | 55 | 428 |  |
|  |  | R: CTAAATGGCTCTGTCGTCTAG |  |  |  |
|  | *fsrC* | F: GTGTTTTTGATTTCGCCAGAGA | 54 | 716 |  |
|  |  | R: TATAACAATCCCCAACCGTG |  |  |  |
|  | *gelE* | F: GGTGAAGAAGTTACTCTGAC | 52 | 704 |  |
|  |  | R: GGTATTGAGTTATGAGGGGC |  |  |  |
|  | *pil* | F: GAAGAAACCAAAGCACCTAC | 53 | 620 |  |
|  |  | R: CTACCTAAGAAAAGAAACGG |  |  |  |
|  | *sprE* | F: CTGAGGACAGAAGACAAGAG | 53 | 432 |  |
|  |  | R: GGTTTTTCTCACCTGGATAG |  |  |  |
| Antibiotic resistance gene | *bla_TEM_* | F: CATTTCCGTGTCGCCCTTAT | 55 | 793 | (4) |
|  |  | R: TCCATAGTTGCCTGACTCCC |  |  |  |
|  | *tetA* | F: GGTTCACTCGAACGACGTCA | 56 | 577 | (4) |
|  |  | R: CTGTCCGACAAGTTGCATGA |  |  |  |

**Supplementary Table S1:** List of primers used to find target genes in this investigation

**Supplementary Table S2:** Pearson Correlation of virulence genes in *E. faecalis* isolated from ornamental birds in Bangladesh

|  | | ***agg*** | ***cyl*** | ***fsrA*** | ***fsrB*** | ***fsrC*** | ***gelE*** | ***sprE*** | ***pil*** | ***ace*** |
| --- | --- | --- | --- | --- | --- | --- | --- | --- | --- | --- |
| ***agg*** | Pearson Correlation | 1 |  |  |  |  |  |  |  |  |
|  | Sig. (2-tailed) |  |  |  |  |  |  |  |  |  |
| ***cyl*** | Pearson Correlation | 0.418^**^ | 1 |  |  |  |  |  |  |  |
|  | Sig. (2-tailed) | 0.005 |  |  |  |  |  |  |  |  |
| ***fsrA*** | Pearson Correlation | -0.024 | -0.184 | 1 |  |  |  |  |  |  |
|  | Sig. (2-tailed) | 0.877 | 0.232 |  |  |  |  |  |  |  |
| ***fsrB*** | Pearson Correlation | 0.102 | 0.091 | 0.203 | 1 |  |  |  |  |  |
|  | Sig. (2-tailed) | 0.509 | 0.558 | 0.187 |  |  |  |  |  |  |
| ***fsrC*** | Pearson Correlation | 0.037 | -0.202 | 0.340^*^ | 0.187 | 1 |  |  |  |  |
|  | Sig. (2-tailed) | 0.812 | 0.188 | 0.024 | 0.224 |  |  |  |  |  |
| ***gelE*** | Pearson Correlation | 0.111 | 0.149 | -0.103 | 0.266 | -0.059 | 1 |  |  |  |
|  | Sig. (2-tailed) | 0.472 | 0.334 | 0.506 | 0.081 | 0.703 |  |  |  |  |
| ***sprE*** | Pearson Correlation | 0.263 | -0.063 | 0.203 | 0.323^*^ | 0.471^**^ | 0.266 | 1 |  |  |
|  | Sig. (2-tailed) | 0.084 | 0.685 | 0.187 | 0.032 | 0.001 | 0.081 |  |  |  |
| ***pil*** | Pearson Correlation | -0.184 | -0.016 | -0.093 | -0.004 | 0.086 | -0.111 | -0.004 | 1 |  |
|  | Sig. (2-tailed) | 0.232 | 0.915 | 0.548 | 0.979 | 0.577 | 0.475 | 0.979 |  |  |
| ***ace*** | Pearson Correlation | -0.016 | -0.293 | 0.056 | -0.113 | -0.086 | -0.069 | 0.385^**^ | 0.036 | 1 |
|  | Sig. (2-tailed) | 0.917 | 0.053 | 0.719 | 0.464 | 0.581 | 0.656 | 0.010 | 0.818 |  |
| **. Correlation is significant at the 0.01 level (2-tailed). | | | | | | | | | | |
| *. Correlation is significant at the 0.05 level (2-tailed). | | | | | | | | | | |

**Supplementary Table S3:** Pearson Correlation of antibiotic resistance patterns in *E. faecalis* isolated from ornamental birds in Bangladesh

|  | | LNZ | AMP | VAN | TEC | E | TE | CIP | NIT | RA | FOS | C |
| --- | --- | --- | --- | --- | --- | --- | --- | --- | --- | --- | --- | --- |
| LNZ | Pearson Correlation | 1 |  |  |  |  |  |  |  |  |  |  |
|  | Sig. (2-tailed) |  |  |  |  |  |  |  |  |  |  |  |
| AMP | Pearson Correlation | .^a^ | .^a^ |  |  |  |  |  |  |  |  |  |
|  | Sig. (2-tailed) |  |  |  |  |  |  |  |  |  |  |  |
| VAN | Pearson Correlation | 0.426^**^ | .^a^ | 1 |  |  |  |  |  |  |  |  |
|  | Sig. (2-tailed) | 0.004 |  |  |  |  |  |  |  |  |  |  |
| TEC | Pearson Correlation | .^a^ | .^a^ | .^a^ | .^a^ |  |  |  |  |  |  |  |
|  | Sig. (2-tailed) |  |  |  |  |  |  |  |  |  |  |  |
| E | Pearson Correlation | 0.396^**^ | .^a^ | 0.235 | .^a^ | 1 |  |  |  |  |  |  |
|  | Sig. (2-tailed) | 0.008 |  | 0.124 |  |  |  |  |  |  |  |  |
| TE | Pearson Correlation | -0.219 | .^a^ | -0.093 | .^a^ | 0.275 | 1 |  |  |  |  |  |
|  | Sig. (2-tailed) | 0.153 |  | 0.547 |  | 0.071 |  |  |  |  |  |  |
| CIP | Pearson Correlation | -0.078 | .^a^ | -0.033 | .^a^ | 0.098 | 0.356^*^ | 1 |  |  |  |  |
|  | Sig. (2-tailed) | 0.614 |  | 0.830 |  | 0.528 | 0.018 |  |  |  |  |  |
| NIT | Pearson Correlation | 0.187 | .^a^ | 0.564^**^ | .^a^ | 0.418^**^ | 0.239 | -0.059 | 1 |  |  |  |
|  | Sig. (2-tailed) | 0.224 |  | 0.000 |  | 0.005 | 0.118 | 0.703 |  |  |  |  |
| RA | Pearson Correlation | 0.239 | .^a^ | 0.235 | .^a^ | -0.092 | -0.173 | -0.141 | 0.022 | 1 |  |  |
|  | Sig. (2-tailed) | 0.118 |  | 0.124 |  | 0.553 | 0.262 | 0.360 | 0.885 |  |  |  |
| FOS | Pearson Correlation | 0.266 | .^a^ | 0.699^**^ | .^a^ | 0.098 | 0.111 | -0.048 | 0.374^*^ | 0.337^*^ | 1 |  |
|  | Sig. (2-tailed) | 0.081 |  | 0.000 |  | 0.528 | 0.472 | 0.759 | 0.012 | 0.025 |  |  |
| C | Pearson Correlation | -0.078 | .^a^ | -0.033 | .^a^ | 0.337^*^ | 0.356^*^ | 0.476^**^ | -0.059 | -0.141 | -0.048 | 1 |
|  | Sig. (2-tailed) | 0.614 |  | 0.830 |  | 0.025 | 0.018 | 0.001 | 0.703 | 0.360 | 0.759 |  |
| **. Correlation is significant at the 0.01 level (2-tailed). | | | | | | | | | | | | |
| *. Correlation is significant at the 0.05 level (2-tailed). | | | | | | | | | | | | |
| a. Cannot be computed because at least one of the variables is constant. | | | | | | | | | | | | |

AMP = Ampicillin, CIP = Ciprofloxacin, C = Chloramphenicol, E = Erythromycin, LEV = Levofloxacin, LZD = Linezolid, NIT = Nitrofurantoin, RA = Rifampin, TE = Tetracycline, TEC = Teicoplanin, VAN = Vancomycin

**References:**

1. European Union Reference Laboratory– Antimicrobial Resistance. Protocol for PCR amplification of E. faecium and E. faecalis recommended by the EURL-AR. 3rd Version. 2014. <https://www.eurl-ar.eu/CustomerData/Files/Folders/21-protocols/281_protocol-for-enterococcus-final-vs3.pdf>.
2. Hashem, Y.A., Amin, H.M., Essam, T.M., Yassin, A.S. and Aziz, R.K., 2017. Biofilm formation in enterococci: genotype-phenotype correlations and inhibition by vancomycin. *Scientific reports*, *7*(1), p.5733.
3. Hashem, Y.A., Yassin, A.S. and Amin, M.A., 2015. Molecular characterization of Enterococcus spp. clinical isolates from Cairo, Egypt. *Indian journal of medical microbiology*, *33*, pp.S80-S86.
4. Randall, L.P., Cooles, S.W., Osborn, M.K., Piddock, L.J.V. and Woodward, M.J., 2004. Antibiotic resistance genes, integrons and multiple antibiotic resistance in thirty-five serotypes of Salmonella enterica isolated from humans and animals in the UK. *Journal of Antimicrobial Chemotherapy*, *53*(2), pp.208-216.
